# Safety of Tattoos, Permanent Make-Up, and Medical Implants in Population-Based 3T Magnetic Resonance Brain Imaging: The Rhineland Study

**DOI:** 10.3389/fneur.2022.795573

**Published:** 2022-03-22

**Authors:** Valerie Lohner, Simon J. Enkirch, Elke Hattingen, Tony Stöcker, Monique M. B. Breteler

**Affiliations:** ^1^Population Health Sciences, German Center for Neurodegenerative Diseases (DZNE), Bonn, Germany; ^2^Clinic for Neuroradiology, University Hospital Bonn, Bonn, Germany; ^3^Department of Neuroradiology, University Hospital Frankfurt, Frankfurt, Germany; ^4^MR Physics, German Center for Neurodegenerative Diseases (DZNE), Bonn, Germany; ^5^Department of Physics and Astronomy, University of Bonn, Bonn, Germany; ^6^Institute for Medical Biometry, Informatics and Epidemiology (IMBIE), Faculty of Medicine, University of Bonn, Bonn, Germany

**Keywords:** epidemiology, MRI safety, population imaging, medical implants, tattoos, permanent make-up

## Abstract

Excluding persons from magnetic resonance imaging (MRI) research studies based on their medical history or because they have tattoos, can create bias and compromise the validity and generalizability of study results. In the population-based Rhineland Study, we limited exclusion criteria for MRI and allowed participants with passive medical implants, tattoos or permanent make-up to undergo MRI. Thereby, we could include 16.6% more people than would have been possible based on common recommendations. We observed no adverse events or artifacts. This supports that most passive medical implants, tattoos and permanent make-up are MRI suitable and can be scanned in research settings.

## Introduction

Magnetic resonance imaging (MRI) is widely used in both clinical practice and research over the past decades. Millions of MRI scans are acquired every year in the US and adverse reactions of medical implants for MRI are rare. The U.S. Food and Drug Administration (FDA) receives only 300 reports on adverse events yearly (1). Most of them describe heating or burns, and projectile accidents by moving objects due to the magnetic field. Based on the potential for heating (2–5), the FDA does not recommend MRI for research purposes for persons with passive devices, including stents, coils, and filters, who cannot provide MRI safety certificates (1). Additional to these guidelines, non-clinical research studies often incorporate other resources to determine the MRI eligibility of passive implants. A powerful online resource to look up MRI eligibility of implants is the website www.mrisafety.com, which provides a comprehensive list of implants and devices with conditional MRI safety information (6). However, in order to look up an implant, the exact type of the implant must be identified first, and not everyone might be aware of what medical device they have been implanted. In clinical practice the presence of medical implants hardly ever poses a problem, since the expected benefit from the imaging procedure outweighs the potential risk for the patient. In non-clinical research settings and especially in studies using high-field MRI, however, such participants are still often excluded as a precaution.

Tattoos and permanent make-up are also a frequent MRI safety concern. Case reports have contributed to the awareness of tattoos being a potential risk in patients undergoing MRI (7–11). However, these case reports might bias the awareness of the potential risks as they do not provide information on the number of persons with tattoos who underwent MRI without adverse events. Although a recent study (*n* = 330) showed that there is only a low risk for adverse reactions in persons with tattoos, this study still excluded persons with larger or neck or head tattoos (12). Another retrospective survey in 135 patients using 1.5 T MRI systems did include tattoos independent of location. They reported tattoo-related adverse events in 1.5% of the patients, which included a slight tingling before the MRI examination started or burning sensation before entering the magnetic field (13).

Whilst safety of a participant in the MRI is of utter importance, stringent eligibility criteria introduce selection bias, which may jeopardize the validity of a study. Together with experts from the field of MR physics, neuroradiology and epidemiology, we investigated whether we could safely broaden eligibility criteria for 3 T MRI examination in a large population-based study, allowing eligible participant with passive medical implants (even without MRI safety certificates), tattoos and permanent make-up to undergo 3 T MRI.

## Materials and Methods

### Study Population

The study is based on the first 5,000 participants of the Rhineland Study, a prospective, single-center, community-based cohort study. We invite all inhabitants aged above 30 years from two geographically defined areas in Bonn, Germany, to participate in the study. The sole exclusion criteria was inability to provide informed consent.

Approval to undertake the study was obtained from the ethics committee of the University of Bonn, Medical Faculty. The study is carried out in accordance with the recommendations of the International Council for Harmonization Good Clinical Practice standards. We obtain written informed consent from all participants in accordance with the Declaration of Helsinki.

### Clarification of MRI Suitability

We established an MRI expert committee that developed the procedure for clarification of MRI suitability. This committee included scientists from Population Health Sciences (VL, MB) and MR Physics (TS) from the DZNE, Bonn, and the Clinic for Neuroradiology (EH, SE), University Hospital Bonn. Depending on the nature of the implants, other experts were consulted (e.g., ophthalmologists, urologists).

**Figure 1:**
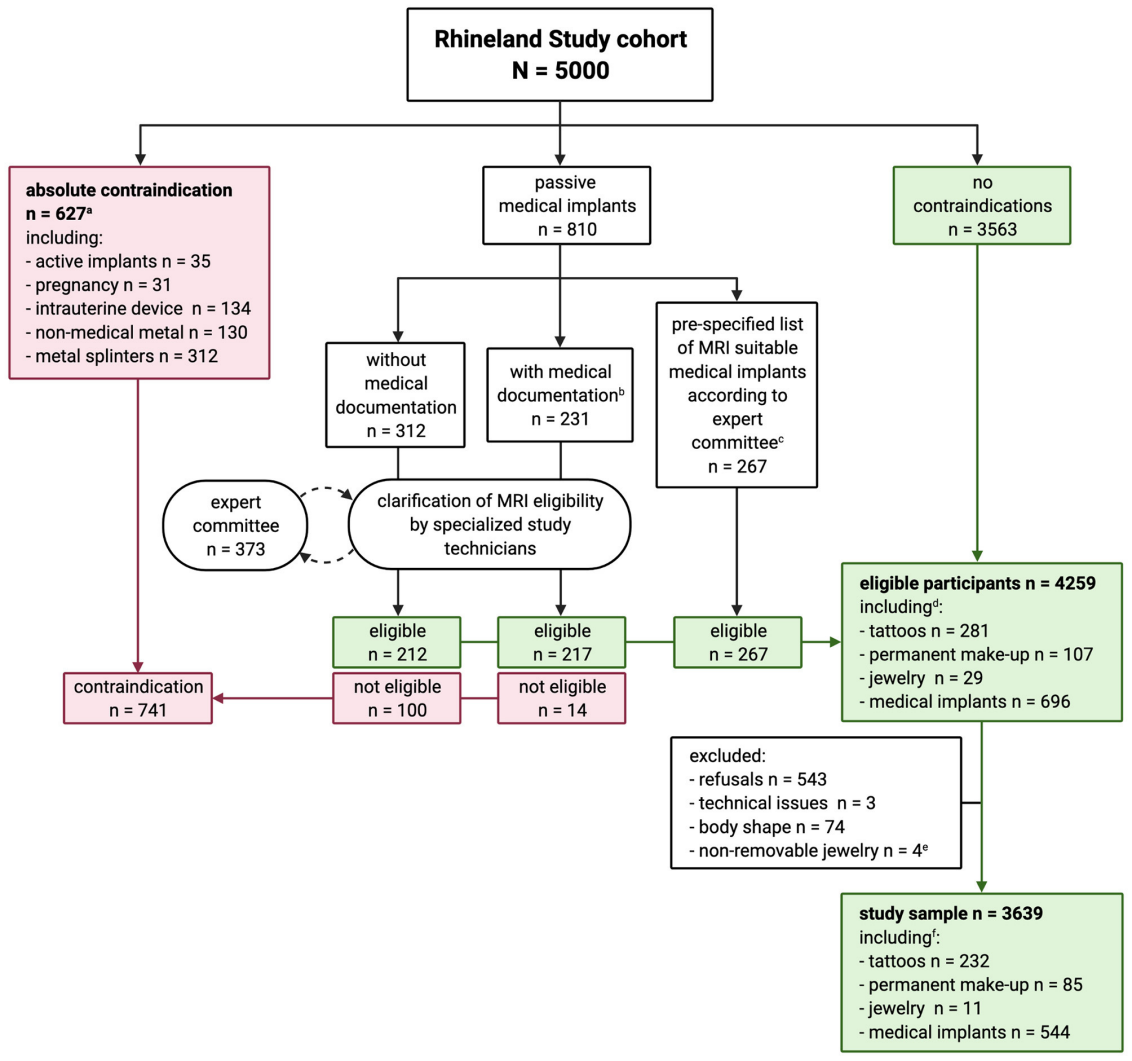
Flowchart of the process of clarification of MRI suitability in the Rhineland Study. ^a^Participant could have more than one absolute contraindication. ^b^Only three participants had MRI safety certificates for their medical implants. ^c^After evaluating our procedure after 1 year, the expert committee considered the following medical implants, if implanted after 2005, as MRI suitable without checking further documentation: hip and knee replacements, stents, bypass, breast implants filled with silicone, and screws, plates and stiffening of the spinal cord < 13 cm. ^d^Three hundred and seventy-six participants had tattoos and/or permanent make-up, of whom 45 also had medical implants. ^e^Participants who were excluded according to stricter exclusion criteria at study start and could not be contacted for reinvitation. ^f^Three hundred and five participants had tattoos and/or permanent make-up, of whom 35 also had medical implants.

Our procedure was as follows (Figure 1): Active implants (e.g., pacemakers), pregnancy, intrauterine devices, non-medical metal and metal splinters were considered absolute MRI contraindications. Tattoos and permanent make-up were not considered contraindications. We did, however, ask for age, size, location, color, and material of the tattoos and permanent make-up. If participants indicated having passive devices, we asked them to bring relevant medical documentation for these (surgery or release reports, implant pass, etc., including age, size and material of the implant). If needed, and with the explicit consent of the participant, we called the hospital which implanted the passive device to ask for further information. Specialized study technicians decided on MRI suitability based on available information, and referred to the MRI expert committee where needed. The expert committee decided on MRI suitability based on current knowledge in both scientific and clinical practice, with the guiding principle to do no harm to participants. In cases of doubt or whenever a possible MRI contraindication could not be ruled out, participants were excluded from MRI.

One year after the introduction of this procedure, the MRI expert committee evaluated it. During this period, 169 participants with medical implants had been discussed by the expert committee and subsequently been scanned without any problems. Based on these experiences, the MRI expert committee made a list of medical implants that from then on could be considered as MRI suitable by the study technicians without further consulting the MRI expert committee. This list included the following medical devices, if implanted after 2005, with or without relevant medical documentation: hip and knee replacements, stents, bypass, clips, breast implants filled with silicone, and screws, plates and stiffening of the spinal cord < 13 cm. The 2005 cut-off was chosen because in recent years such implants are typically made of titanium. A medical implant had to be implanted at least 6 weeks before the MRI examination.

### MRI Data Acquisition

All eligible participants underwent a one-hour MRI examination of brain structure and function on 3 T MRI scanners (Siemens Prisma Magnetom, Erlangen, Germany). The scanners were equipped with an 80 mT/m gradient system and a 64-channel phased-array head-neck coil. All MRI sequences and protocols were either developed in-house for the purpose of the Rhineland Study or based on Siemens product sequences. The MRI protocol included the following sequences: a 3D T1-weighted Multi-Echo Magnetization Prepared RApid Gradient-Echo sequence [ME-MPRAGE, acquisition time (TA) = 6.5 min, time of repetition (TR) = 2,560 ms, inversion time (TI) = 1,100 ms, flip angle 7°, field of view (FOV) = 256 × 256 mm, 0.8 mm isotropic] (14, 15), a 3D T2-weighted Turbo-Spin-Echo (TSE) sequence [TA = 4.6 min, TR = 2,800 ms, echo time (TE) = 405 ms, FOV = 256 × 256 mm, 0.8 mm isotropic] (16, 17), a 3D T2 FLuid-Attenuated Inversion Recovery (FLAIR) sequence [TA = 4.5 min, TR = 5,000 ms, TE = 393 ms, TI = 1,800 ms, FOV = 256 × 256 mm, 1.0 mm isotropic], a motion robust quantitative susceptibility weighted (QSM) sequence based on a 2D-segmented 3D gradient echo-planar imaging (EPI) sequence using multiple echo times (6 echo times, TA = 5.7 min, TR = 32 ms, flip angle 14°, FOV = 212 × 212 mm, 0.8 mm isotropic) (18), for simultaneous-multi-slice diffusion weighted MRI (dMRI), a spin-echo echo-planar imaging (SE-EPI) sequence applying threefold slice-acceleration and a compressed-sensing diffusion spectrum imaging protocol (TA = 11.4 min, TR = 5,500 ms, TE = 105 ms, band width 1,624 Hz/Px, FOV = 210 × 210 mm, 1.5 mm isotropic) (19–22), a 3D EPI sequence using 2D Controlled Aliasing In Parallel Imaging Results IN Higher Acceleration (CAIPIRINHA) sampling with variable echo train lengths, rapid water excitation and fat-selective inversion recovery was applied to collect resting-state fMRI data (TA = 10.5 min, TR = 570 ms, TE = 30 ms, TI = 240 ms, flip angle 16°, FOV = 216 × 216 mm, 2.4 mm isotropic) (23), and abdominal MRI was performed for 72 axial slices centered in the middle of the third lumbar vertebra using a breath-hold two-point Dixon sequence while the participants were in supine position with arms placed at side (2 echo times, TA = 0.2 min, TR = 4.12 ms, flip angle 6°, FOV = 500 × 437 mm, resolution 2.0 × 2.0 × 5.0 mm). The T1- and T2-weighted sequences employed twofold parallel imaging acceleration using CAIPIRINHA and elliptical sampling (24, 25).

Before the MRI examination, we verbally informed all participants with medical implants, non-removable jewelry, tattoos and/or permanent make-up about the possibility of adverse events, including tingling sensations, (slight) heating, and burning. They were instructed to squeeze the alarm ball during the MRI examination as soon as they would feel any tingling sensation. In case of an adverse reaction, we would ask about their symptoms and document these as well, and provide first aid if needed.

For participants with head implants or permanent make-up, we checked all scouts for possible artifacts which would require immediate stopping of MRI data acquisition. For permanent make-up, this would include any artifacts on the scouts, for head implants any artifact that would make the scan of the brain unreadable. Additionally, all T1-weighted, T2-weighted, and FLAIR scans have been visually inspected for quality during the initial quality assessment of the Rhineland Study, where two raters independently checked for artifacts that might affect the quality of automated brain segmentations.

### Sample Size and Minimum Detectable Effect

We have calculated the proportion of adverse events that we could have detected with 90% and 80% confidence given our sample size of people with tattoos or medical implants (*n* = 305 and *n* = 544, respectively) (26).

## Results

Figure 1 gives an overview of MRI suitability in the Rhineland Study. Of the 5,000 participants, 3,563 (71.3%) had no contraindications, 627 (12.5%) had an absolute contraindication, and 810 (16.2%) had a passive medical implant. We ultimately deemed 696 (85.9%) of the passive medical implants MRI suitable. The expert committee discussed 373 cases and considered 352 of those as MRI suitable. We excluded participants who could not provide enough information to assess suitability.

In total, 4,259 (85.2%) participants were considered eligible for MRI, of whom 3,639 (85.4%) were actually scanned [mean age 54.7 (SD = 13.7) years, 57.8% women (Table 1)]. Of those we scanned, 544 (14.9%) had passive medical implants, 305 (8.4%) had either tattoos (6.4%), permanent make-up (2.3%), or both (0.3%), 35 (1.0%) had medical implants and tattoos, and 11 had non-removable jewelry (wedding rings, piercings).

**Table 1:** Characteristics of the participants of the Rhineland Study who underwent MRI.

		**Rhineland Study cohort (*n* = 3,639)**
Age in years [mean (SD)]		54.7 (13.7)
Women [No. (%)]		2103 (57.8)
Passive medical implants [No. (%)]		544 (14.9)
Non-removable jewelry [No. (%)]		11 (0.3)
Tattoo and/or permanent make-up [No. (%)]^a^		305 (8.4)
Tattoo [No. (%)]		232 (6.4)
Permanent make-up [No. (%)]		85 (2.3)
Total tattoo size in cm^2^ [median (IQR)]		100 [30–450]
Number of individual	1	181 (59.3)
tattoos/permanent make-up [No. (%)]	2	64 (21.0)
	3	35 (11.5)
	4	9 (3.0)
	5	9 (3.0)
	6	1 (0.3)
	7	1 (0.3)
	8	3 (1.0)
	Unknown^b^	1 (0.3)
	Disappeared^c^	1 (0.3)
Body location [No. (%)]	Arm	109 (20.5)
	Eyebrows	51 (9.6)
	Eyelid	56 (10.5)
	Foot	19 (3.6)
	Hand	7 (1.3)
	Lips	11 (2.1)
	Lower leg	49 (9.2)
	Neck	12 (2.3)
	Private parts	2 (0.4)
	Torso	180 (33.8)
	Unknown	2 (0.4)
	Upper leg	28 (5.3)
	Wrist	6 (1.1)

a *This includes 12 participants who had both tattoos and permanent make-up*.

b *No information provided by participant*.

c *Tattoo was not visible anymore after 2 years*.

**Figure 2:**
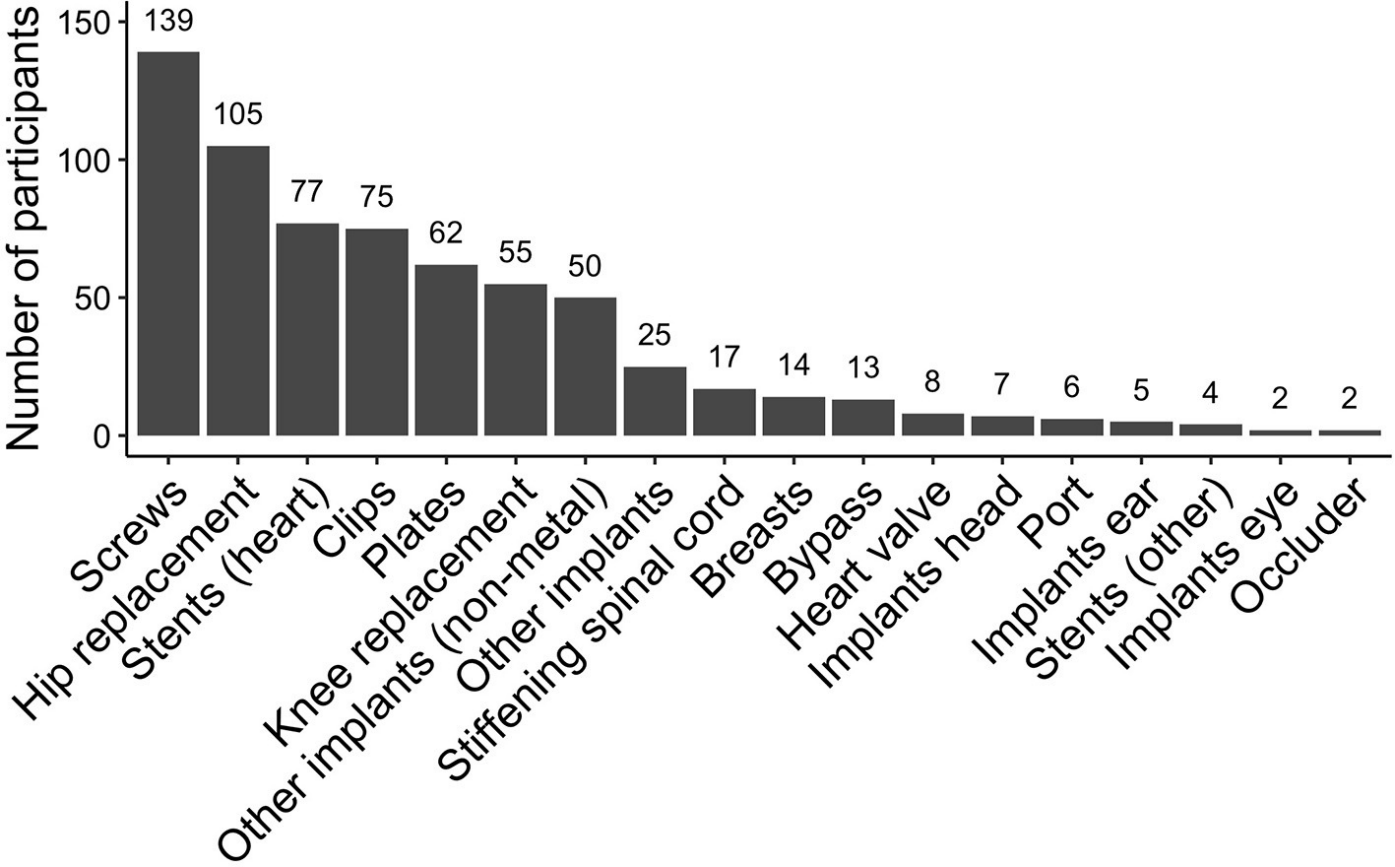
Frequency of eligible medical implants that were scanned at 3T in the Rhineland Study. Participants could have multiple plates or screws, these were each counted as one implant. *Other implants* included: wire cerclage, threads made from titanium, patches made from Teflon, urinary tract implants, broken dental files. *Other implants (non-metal)* included: hernia mesh, neobladder, artificial bone mass, gastric band.

Participants had up to six medical implants, mostly plates, screws, stents, clips, or hip- or knee-replacement (Figure 2), which were up to 48 years old with a median age of 7 years [interquartile range (IQR): 3–13 years].

Among participants with tattoos, the number of tattoos (including permanent make-up) per person ranged from one to eight, at in total 532 individual body locations. Most frequent locations were on the torso (33.8%), arms (20.5%), or legs (lower leg: 9.2%, upper leg: 5.3%) (Table 1). The majority of the tattoos (78.8%) was located above the waist and hence within the main or fringe field of the radiofrequency transmitting body coil and the gradient coils of the MRI scanner, 21.2% were located in the head coil. Tattoos and permanent make-up were between 1 and 41 years old, with a median age of 10 years (IQR: 4–20 years). Median size was 100 cm^2^ (IQR: 30–450 cm^2^), ranging up to 7,960 cm^2^, with 72 (17.7%) tattoos being larger than 20 cm in dimension (Table 1). We scanned 24 participants with tattoos covering more than 5% of the mean sex-specific body surface area (27). Most tattoos were mono-colored (64.3%), most used colors were black (52.1%), brown (5.5%), black-red (4.9%), black-blue (4.5%), black-red-green (2.4%). Most participants were not aware of the material of the tattoo (73.2%), only 2.2% reported that it was tattoo ink that did not contain any metal, 1.2% reported that their tattoo was self-made, and 1.0% did not know the material of their tattoo, but spontaneously reported that they got it outside of Europe or the USA.

None of the participants reported adverse events nor was the quality of any of the MR scout images reduced by any implants or permanent make-up. There were no artifacts seen during the initial quality assessment due to permanent make-up or medical implants in the head which made the brain images unreadable.

### Comparison to Previous Recommendations

With regard to tattoos, if we had followed the procedure from a recent study on MRI safety of tattoos, we would have had to exclude 182 of 376 participants who we considered eligible, because of tattoo location (head: *n* = 108, neck: *n* = 15, genital area: *n* = 2), tattoos covering more than 5% of the total body area (*n* = 28), tattoos bigger than 20 cm in diameter (*n* = 60), or tattoos <20 cm apart from each other (*n* = 21) (multiple reasons possible) (12).

If we had followed most recent recommendations by the FDA that require an MRI safety certificate (1), we would have had to exclude all but 3 participants for their medical implant (807 of 810 participants). Following our procedure, we only excluded 114 of 810 participants, yielding an additional 693 eligible participants.

Thus, compared to these established practices and FDA guidelines we classified an additional 830 participants with tattoos or medical implants (45 had both) as MRI eligible (16.6% of our source population). Of these, 703 participants underwent MRI.

Of note, the FDA guidelines can be interpreted more loosely, allowing for an implant to be identified as MRI suitable based on other medical documentation. Had we used those criteria, we still would have had to exclude 589 of our 810 participants with passive medical implants.

### Sample Size

With our given sample size for tattoos and medical implants, we would be able to detect with 90% confidence adverse reactions in 0.8 and 0.4%, respectively, and with 80% confidence in 0.5 and 0.3%, respectively.

## Discussion

In this large population-based study, we allowed participants with passive medical implants without MRI safety certificates, tattoos, or permanent make-up to undergo 3 Tesla MRI. We did not observe any adverse events or artifacts that notably reduced quality of the brain scans. Through our relaxed MRI eligibility criteria, we could include 16.6% more people than would have been possible based on FDA guidelines (1) and recommendations from a previous study (12).

Older case reports described adverse reactions in people with tattoos undergoing MRI (7–11), yet a more recent study in 330 persons reported that the probability of having a tattoo-related adverse reaction was only 0.17% (12). However, that study excluded participants with tattoos on head, neck, or genital area, bigger than 20 cm in diameter, not 20 cm apart from each other, and covering more than 5% of the total body area, because of fear of adverse reactions. In a retrospective survey among in 135 persons with tattoos including head and neck tattoos who underwent clinical MRI, 1.5% reported adverse reactions before the actual MRI scanning, which, however, were not long-lasting (13). In our study, we included all persons with tattoos and permanent make-up regardless of size or location. None of the participants reported any adverse events.

The FDA recommends to exclude people from MRI for research purposes if their medical implant cannot be identified as MRI eligible (1). Of course, most studies do not solely base their guidelines for MRI eligibility on the FDA recommendation, but rather on a combination of resources, including expert knowledge or websites such as www.mrisafety.com. Nevertheless, it is essential to be able to identify medical implants in order to confirm eligibility. We found that <0.5% of those with a passive medical implant had an MRI safety certificate. Most of our participants had no relevant documentation to identify the medical implant, and would therefore have been excluded had we strictly followed the FDA recommendations. We were able to classify two thirds of these participants as MRI eligible, based on information the participant provided verbally. In the excluded cases, participants could not tell us what exact procedures they underwent nor when. Therefore, we could not out rule any potential risks for the participant to undergo MRI.

Our approach emphasizes the importance of MRI expert panels involved in the clarification of MRI eligibility. Due to the combined knowledge on clinical and physical MRI, we were able to increase the number of participants undergoing MRI. We propose that new (population-based) research studies establish MRI expert panels to determine MRI safety of passive devices, incorporating recent advances in the scientific communities [e.g., ISMRM (ISMRM & SMRT MR Safety Resources^1^), www.mrisafety.com (6)] as well as clinical practices, thereby reducing selection bias in research studies.

Here, we defined adverse reactions as pressing the alarm ball during the MRI examination. Previous studies have asked participants afterwards about their experience in the MRI. We refrained from doing so since we instructed our participants extensively before entering the scanner to press the alarm ball whenever something would feel off.

A limitation of our study is that only 24 of our scanned participants had tattoos covering more than 5% of the total body area. Although we asked participants about the material of their tattoos, most of them did not know. Unfortunately, we did not specifically ask for the country where the tattoos had been made. Additional studies are therefore required to investigate the MRI suitability of full-body tattoos, and preferably including information on country where and material with which the tattoos were done. While we visually checked the brain scout for artifacts in participants with head implants and permanent make-up at the beginning of the MRI examination, we did not use automated metrics or quantitative assessments for this. However, there were no artifacts that made the images unreadable.

## Conclusion

We conclude that most passive medical implants (even without MRI safety certificates), tattoos, and permanent make-up are eligible for 3 Tesla MRI research studies. Our procedure could guide new research studies in the clarification of MRI suitability. This is crucial to reduce selection bias in, and thereby increase generalizability and validity of, MRI research studies.

## Data Availability Statement

The datasets presented in this article are not readily available because of data protection regulations. Access to data can be provided to scientists in accordance with the Rhineland Study's Data Use and Access Policy. Requests to access the datasets should be directed to Rhineland Study's Data Use and Access Committee, RS-DUAC@dzne.de.

## Ethics Statement

The studies involving human participants were reviewed and approved by University of Bonn, Medical Faculty. The participants provided their written informed consent to participate in this study.

## Author Contributions

VL, EH, TS, and MB contributed to conception and design of the study. VL performed the statistical analysis and wrote the first draft of the manuscript. All authors contributed to data acquisition and analysis, manuscript revision, and read and approved the submitted version.

## Funding

The Rhineland Study at the DZNE is predominantly funded by the Federal Ministry of Education and Research (BMBF) and the Ministry of Culture and Science of the German State of North Rhine-Westphalia.

## Conflict of Interest

The authors declare that the research was conducted in the absence of any commercial or financial relationships that could be construed as a potential conflict of interest.

## Publisher's Note

All claims expressed in this article are solely those of the authors and do not necessarily represent those of their affiliated organizations, or those of the publisher, the editors and the reviewers. Any product that may be evaluated in this article, or claim that may be made by its manufacturer, is not guaranteed or endorsed by the publisher.

## Footnotes

^1^Available online at: https://www.ismrm.org/mr-safety-links/ (accessed January 27, 2022).
